# Urinating Standing versus Sitting: Position Is of Influence in Men with Prostate Enlargement. A Systematic Review and Meta-Analysis

**DOI:** 10.1371/journal.pone.0101320

**Published:** 2014-07-22

**Authors:** Ype de Jong, Johannes Henricus Francisca Maria Pinckaers, Robin Marco ten Brinck, Augustinus Aizo Beent Lycklama à Nijeholt, Olaf Matthijs Dekkers

**Affiliations:** 1 Department of Urology, Leiden University Medical Center (LUMC), Leiden, the Netherlands; 2 Department of Clinical Epidemiology, Leiden University Medical Center (LUMC), Leiden, the Netherlands; University of York, United Kingdom

## Abstract

**Background:**

It is suggested that the body posture during urination can influence urodynamic parameters in patients with Lower Urinary Tract Symptoms (LUTS) to an extent approaching pharmacological interventions. In this article, the influence of body position during micturition on maximum urinary flow rate (Qmax), voiding time (TQ) and post-void residual volume (PVR) in healthy males and patients with LUTS is analyzed by means of a systematic review and meta-analysis.

**Evidence Acquisition:**

A systematic search was conducted in 14 medical databases. Studies comparing urodynamic parameters in standing versus sitting position were eligible for inclusion. Studies were stratified according to health status of included male participants: healthy individuals and patients with LUTS. Standardized mean differences for Qmax, TQ and PVR were pooled in a random effects model.

**Results:**

Eleven articles were included. In men with LUTS, a significantly lower PVR (−24.96 ml; 95%CI −48.70 to −1.23) was shown in sitting position compared to standing. In accordance, Qmax was increased (1.23 ml/s; 95%CI −1.02 to 3.48), and TQ was decreased (−0.62 s; 95%CI −1.66 to 0.42) in sitting position, although these differences did not reach statistical significance. In healthy men, Qmax (0.18 ml/s; 95% CI −1.67 to 2.02), TQ (0.49 s; 95%CI −3.30 to 4.27) and PVR (0.43 ml; 95%CI −0.79 to 1,65) were similar in sitting and standing position.

**Conclusion:**

For healthy men, no difference is found in any of the urodynamic parameters. In patients with LUTS, the sitting position is linked with an improved urodynamic profile.

## Introduction

Ever since men had the choice to urinate either standing or sitting, the optimal voiding position has been a topic of discussion. The introduction of the modern flush toilet during the 19th century [1] may have intensified this discussion. Geographically, voiding positions differ. In most Western countries the standing position is common, while in Eastern and Asian countries the sitting and crouching positions are more common [2]–[8]. The first medical description of the influence of voiding position on bladder health dates from 1883, when the English medical officer Raglan W. Barnes [9] stated his concerns about the high prevalence of bladder stones in the Indian population, which he linked to their voiding position. However, Barnes is likely to be biased as he perceived himself morally superior to the native population, which can be concluded from his last statement: “as the march of civilisation proceeds in India, he [the native] may become morally and physically more upright.”

Barnes' hypothesis that the voiding position could influence urodynamic parameters to such an extent that changes therein can lead to urological diseases is intriguing and may be relevant for the most prevalent group of urologic diseases: Lower Urinary Tract Symptoms (LUTS). Benign Prostate Hyperplasia (BPH), a nonmalignant enlargement of the prostate with an age-related prevalence of up to 90%, most commonly causes LUTS in males [10]. The urodynamic profile of LUTS is characterized by a decreased maximum urinary flow rate (Qmax, ml/s), an increased voiding time (TQ, s) and post-void residual volume (PVR, ml), which may result in complaints and complications like cystitis or bladder stones. Standard clinical management of LUTS therefore aims to decrease PVR and TQ while increasing Qmax [5], [8], [11]–[15], which can be reached pharmacologically with use of alpha-blockers and 5α-reductase inhibitors. This form of treatment however only shows modest alleviation of the symptoms [16]. An alternative treatment is surgery, for example in the form of transurethral resection of the prostate (TURP) [13], [17].

Since Barnes propagated his hypothesis, only a handful of studies have investigated the effects of voiding posture on urodynamic parameters by comparing the standing versus the sitting position. One author [18] suggested that changing one's voiding position may yield in an effect that can approach the effects of standard pharmaceutical management. However, due to the heterogeneity of results in these studies, no conclusion can be drawn without performing a meta-analysis. In this article, we summarize the evidence of an easy lifestyle change in addition to the standard therapy: changing ones voiding position in order to achieve a beneficial urodynamic profile. This meta-analysis aims to analyze the influence of body position on urodynamic parameters in both healthy males and male patients with LUTS.

## Methods

We have conducted this review in accordance with the PRISMA guidelines [19]; this checklist is provided in Table S1. No protocol was defined beforehand.

### Data sources and search strategy

To identify eligible studies, we applied a systematic literature search to 14 electronic databases: PubMed, Embase (OVID-version), PubMed Central, Web of Science, the Cochrane Library, CINAHL, PsycINFO, Academic Search Premier, ScienceDirect, SpringerLink, Wiley Online Library, Lippincott-Williams&Wilkins (Journals@Ovid Full Text), Highwire, and Google Scholar. A non-systematic, manual search was conducted in the WHO International Clinical Trials Registry Platform. The searches were performed on April 25th, 2013.

Search queries were created in cooperation with a medical librarian. We combined synonyms for “position”, “standing”, and “sitting” with synonyms for “urinating”, “urodynamics”, and “urination disorders”. The search was restricted to human studies and male subjects. No restrictions were set to language or publication year. Complete search queries for each database are shown in Appendix S2. Non-English articles were translated if necessary. Only studies published as full-text articles were considered for meta-analysis. In order to obtain more information or full-text articles, affiliated authors were contacted via email or telephone.

### Study selection

Search results from different databases were combined and duplicates were removed using EndNote for Windows (version X6, Thomson Reuters, 2012) and Reference Manager (version 12, Thomson Reuters 2008). We considered studies to be eligible for inclusion if they compared standing voiding position with the sitting in males and measured at least one of the three outcome measures of interest: Qmax, TQ, and PVR. Studies in healthy men as well as in men with clinical LUTS were considered for inclusion. Studies with conditions other than LUTS or with participants under the age of 18 were not eligible. Likewise, articles with no analyzable data or unavailability of the full text were not included.

Three reviewers (DJ, P, and TB) independently reviewed all citations and selected eligible studies. In three meetings, consensus was sequentially reached based on title, abstract, and full-text. In case of disagreement, an expert in the field of urology was consulted (LaN). A snowball search of the reference lists of included articles was performed independently; eligibility was assessed using the same method.

### Data extraction and risk of bias assessment

A data extraction form was designed which was adapted after piloting. Data were extracted independently; inconsistencies in the data were discussed and resolved. We extracted data on: study design, year of publication, sample size, patient characteristics (age and morbidity), studied urodynamic parameters (Qmax, TQ, and PVR), and urination positions. Relevant missing information was requested from the study authors.

We assessed the studies on their risk of bias concerning variables known to be of influence in urodynamic research. Studies were considered to harbor a high risk of bias in the case of (1) inadequate exposure determination, (2) inadequate assessment of outcomes and (3) inadequate standardization of voiding conditions. For adequate exposure determination, studies should have assessed the severity of LUTS by a standardized questionnaire (International Prostate Symptom Score, IPSS [20]). As all included studies used a one-group (cross-over) study design, differences in baseline characteristics were not an issue. For adequate outcome assessment, total bladder capacity should have been measured and the technique used for the assessment of urodynamic parameters should have been described. To ensure a valid comparison between standing and voiding position, voiding conditions should have been standardized and the following variables should have been taken into account: the influences of (1) the setting for the measurements (in a private, non-observed clinical setting, in an observed clinical setting or at home [21]), (2) the circadian rhythm [22], (3) the time since last ejaculation [23] and (4) defecation [5], [18], (5) changes in intra-abdominal pressure [24], [25], and (6) the accustomed voiding position [6], [8], [11], [14], [18], [26]–[28] on urodynamics.

### Statistical analysis

The primary outcomes of our study were pooled mean differences of Qmax, TQ, and PVR. As all studies reported these outcomes on the same scale, no standardization was necessary. We aimed to extract mean differences and accompanying standard errors based on paired t-tests. If these data were not provided, we extracted the mean and standard error for sitting and standing position separately. Subsequently, we calculated a mean difference with a combined standard error according to the Cochrane Handbook for Systematic Reviews of Interventions using a correlation coefficient of 0.5 [29], accounting for the fact that data were paired. Four articles [3], [7], [30], [31] were assumed to report standard errors instead of standard deviations and data were subsequently converted accordingly by multiplying the value with the square root of number of participants. One study [32] reported interquartile ranges which were converted to standard deviations by multiplying the value by 0.68, hereby assuming normal distribution.

Study results were stratified by the included population (healthy participants and participants with LUTS). Mean differences were pooled in a random effects model. I^2^ statistics were calculated as a measure of between-study heterogeneity. Analysis of the data was performed with STATA (version 12.0, STATA Corp).

## Results

**Figure 1:**
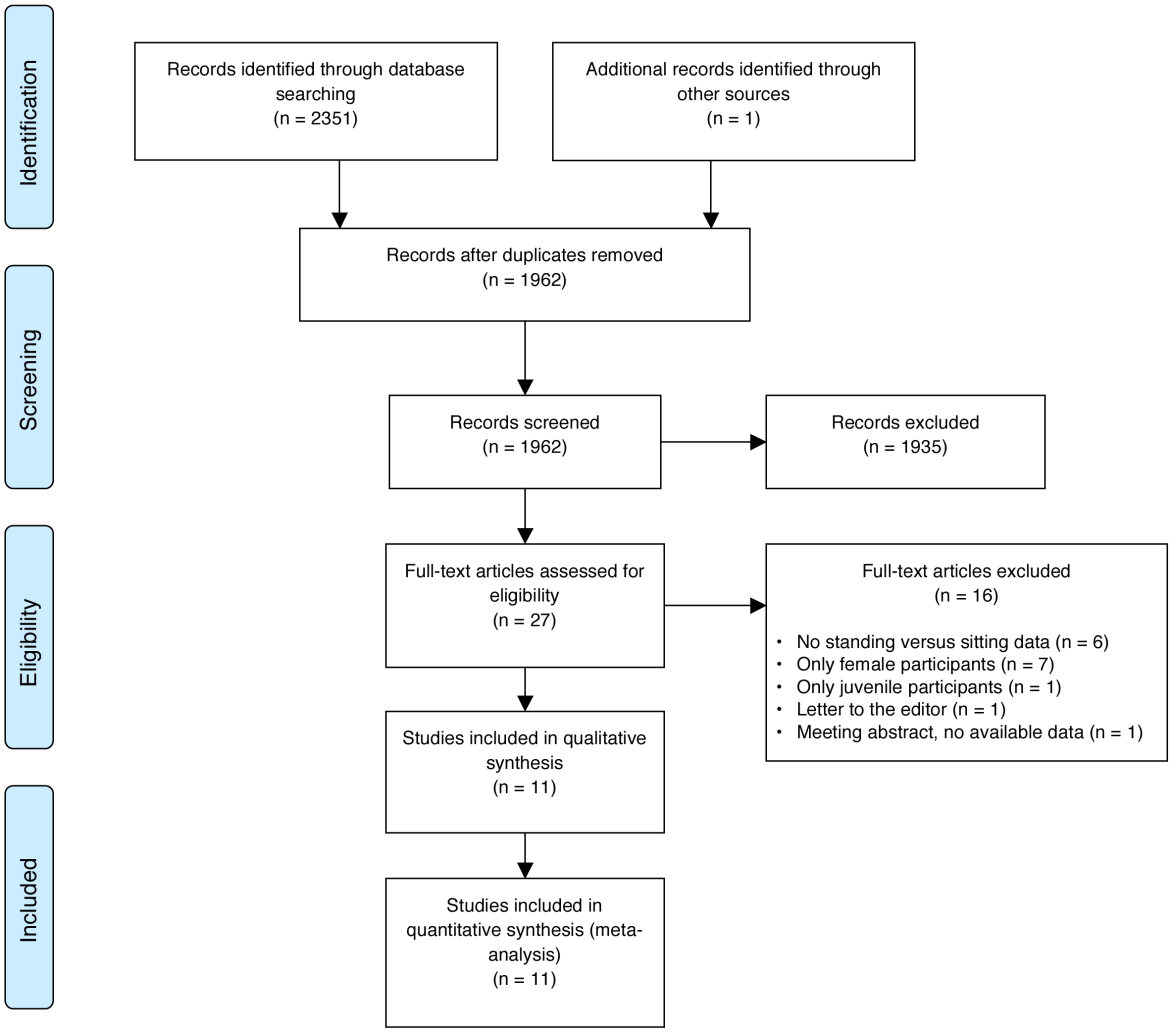
PRISMA flowchart showing the study selection process.

Overall, 2352 publications were retrieved through our search strategies. A flowchart of the study selection is presented in figure 1. After removal of duplicates, 1962 publications were independently assessed for eligibility. A total of 69 abstracts were identified, 27 relevant publications were assessed in full-text for eligibility. Eleven studies [3], [7], [8], [14], [18], [26], [28], [30]–[33] fulfilled the inclusion criteria and were included in the meta-analysis (table 1). A snowball search of the references in these 11 articles did not yield additional articles. One author [18] sent his database for further analysis.

**Table 1:** Characteristics of included studies.

Study source	Participants	Mean difference in sitting versus standing posture (95% CI)
**Aghamir et al, 2005, Iran**	N = 20, stratified:	Group A:
	Group A: n = 10 healthy men	Qmax 0.50 (−1.55 to 2.55)
	Group B: n = 10 LUTS/BPH patients	TQ −2.50 (−4.79 to 0.21)
		PVR n/a
		Group B:
		Qmax 0.20 (−0.92 to 1.32)
		TQ −6.50 (−20.33 to 7.33)
		PVR −63.00 (−96.58 to −29.42)
**Amjadi et al, 2011, Iran**	N = 31 healthy men	Qmax 1.00 (−2.17 to 4.17)
		TQ −1.80 (−5.65 to 2.05)
**Choudhury et al, 2010, India**	N = 61 healthy men	Qmax −4.00 (−5.90 to −2.10)
		TQ 9.30 (4.84 to 13.76)
		PVR 1.50 (0.04 to 2.96)
**El-Bahnasawy et al, 2008, Egypt**	N = 200 LUTS/BPH patients	Qmax 0.70 (−0.37 to 1.77)
		TQ −2.50 (−7.22 to 2.22)
		PVR −13.10 (−24.00 to −2.20)
**Eryıldırım et al, 2008, Turkey**	N = 30 healthy men	Qmax 4.50 (2.04 to 6,96)
		TQ −1.40 (−3.04 to 0.24)
		PVR −0.20 (−3.35 to 2.95)
**Koc et al, 2006, Turkey**	N = 110 LUTS/BPH patients, stratified:	Both groups:
	Group A: N = 44 (alpha blockers)	Qmax 1.80 (0.76 to 2.84)
	Group B: N = 66 (control group)	TQ −2.50 (−7.31 to 2.31)
		PVR −3.40 (−18.71 to 11.91)
**Norg et al, 2009, Netherlands**	N = 20 LUTS/BPH patients	Qmax −0.55 (−1.08 to −0.02)
		TQ −0.38 (−1.48 to 0.72)
**Salem et al, 2009, Egypt**	N = 100 LUTS/BPH patients	Both groups:
		Qmax 5.90 (5.12 to 6.68)
		PVR −55.70 (−70.47 to −40.93)
**Ünsal et al, 2004, Turkey**	N = 88, stratified:	Group A:
	Group A: N = 44 (healthy men)	Qmax 0.50 (−1.04 to 2.04)
	Group B: N = 44 (LUTS/BPH patients)	PVR −1.20 (−3.38 to 0.98)
		Group B:
		Qmax −0.70 (−1.72 to 0.32)
		PVR 3.30 (−19.80 to 26.40)
**Ünsal et al, 2004, Turkey**	N = 36 healthy men	Qmax 0.71 (−0.64 to 2.06)
		PVR 0.60 (−1.33 to 2.53)
**Yamanishi et al, 1999, Japan**	N = 21 healthy men	Qmax −2.10 (−5.77 to 1.57)

**Table 1** Characteristics of included studies for the difference between the urodynamic parameters: maximum urinary flow rate (ml/s, Qmax), voiding time (s, TQ), and post-void residual volume (ml, PVR) in the sitting versus standing position in healthy males and male patients with Lower Urinary Tract Symptoms (LUTS).

### Study characteristics

Included studies were published between 1999 and 2012. All studies used a cross-over design. A total of 800 participants were included, with the number of participants in individual studies ranging from 20 to 200. In five studies [3], [7], [26], [31], [33] only healthy men were included, in four studies [8], [14], [18], [28] only patients with LUTS, and in two studies [30], [32] both groups were studied. For studies with healthy participants all seven [3], [7], [26], [30]–[33] investigated Qmax, four measured TQ [3], [7], [26], [31], and four studies [3], [26], [30], [31] measured PVR. For studies with LUTS patients, all six [8], [14], [18], [28], [30], [32] investigated Qmax, four studies [14], [18], [28], [32] measured TQ, and five studies [8], [14], [28], [30], [32] measured PVR. The study characteristics are shown in table 1.

### Risk of bias assessment

Risk of bias assessment is presented in table 2. Of the six LUTS studies, two [14], [30] defined the severity of LUTS by means of the IPSS and clinical examination, two [8], [28] by clinical examination only, one [18] by IPSS only, whereas one study [32] did not describe the severity at all. With regard to standardization of measurements, seven studies [3], [7], [14], [28], [30], [31], [33], used a private, non-observed clinical setting, one [18] instructed to measure at home, and three studies [8], [26], [32] did not describe the setting in which measurements took place. The following factors of influence were accounted for: circadian rhythm in three studies [3], [14], [26], time since last ejaculation in none and time since last defecation in one [18] of the studies, intra-abdominal pressure in six studies [3], [8], [28], [30], [31], [33], and the accustomed position in two studies [18], [26]. With regard to outcome assessment, of eight [3], [8], [14], [26], [28], [30]–[32] studies which measured PVR only two [8], [28] measured total bladder capacity. All studies defined their methods for the other urodynamic measurements.

**Table 2:** Risk of bias assessment.

		Exposure determination		Assessment of outcomes			Standardization of voiding conditions					
		Age	LUTS-symtoms	PVR	TBC	Other >measurements	Setting	Circadian rhythm	Ejaculation	Defecation	IAP	Preferred position
**LUTS**	**El-Bahnasawy et al, 2008, Egypt**	Yes	Clinical	Yes	Yes	Yes	Clinical and private‡	No	No	No	Yes	No
	**Koc et al, 2012, Turkey**	Yes	Clinical and IPSS	Yes	No	Yes	Clinical and private‡	Yes	No	No	No	No
	**Norg et al, 2009, Netherlands**	Yes	Clinical	NA^†^	NA^†^	Yes	Home	No	No	Yes	No	Yes
	**Salem et al, 2009, Egypt**	Yes	No	Yes	Yes	Yes	No	No	No	No	Yes	No
**Both**	**Aghamir et al, 2005, Iran**	Yes	Clinical	No	No	Yes	No	No	No	No	No	No
	**Ünsal et al, 2004, Turkey**	Yes	IPSS	Yes	No	Yes	Clinical and private‡	No	No	No	Yes	No
**Healthy**	**Amjadi et al, 2011, Iran**	Yes	NA*	NA^†^	NA^†^	Yes	Clinical and private‡	No	No	No	No	No
	**Choudhury et al, 2010, India**	Yes	NA*	Yes	No	Yes	No	Yes	No	No	No	Yes
	**Eryıldırım et al, 2006, Turkey**	Yes	NA*	Yes	No	Yes	Clinical and private‡	Yes	No	No	Yes	No
	**Ünsal et al, 2004, Turkey**	Yes	NA*	Yes	No	Yes	Clinical and private‡	No	No	No	Yes	No
	**Yamanishi et al, 1999, Japan**	Yes	NA*	NA^†^	NA^†^	Yes	Clinical and private‡	No	No	No	Yes	No

**Table 2** Assessment of the risk of bias in individual studies based on (1) exposure determination which includes standardization of age and assessment of LUTS severity by IPSS questionnaires, (2) assessment of study outcomes, which concerns standardization of measurements and (3) standardization of voiding conditions, including the influence of the setting, circadian rhythm, time since last ejaculation and defecation, and instructions about intra-abdominal pressure. LUTS: Lower Urinary Tract Symptoms, PVR: Post-void residual volume, TBC: Total bladder capacity, IAP: intra-abdominal pressure, IPSS: International Prostate System Score. *No LUTS patients were included in this study. †No PVR measurements and subsequently total bladder capacity measurements were performed in this study. ‡Measurements took place in a private setting, i.e. out of sight and hearing range of researchers.

### Meta-analysis of urodynamic parameters in healthy individuals

**Figure 2:**
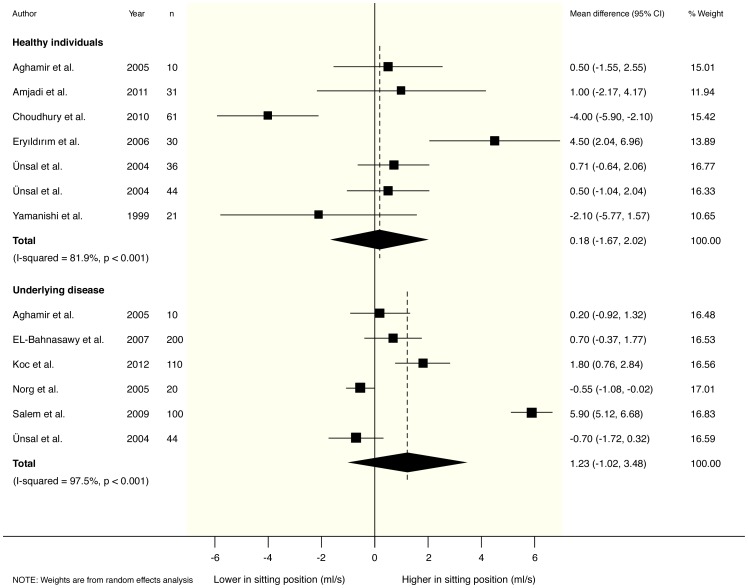
Forest plot from random effects meta-analysis on the difference in maximum urinary flow rate (Qmax) in both healthy males and male patients with Lower Urinary Tract Symptoms (LUTS) in the sitting versus standing position.

**Figure 3:**
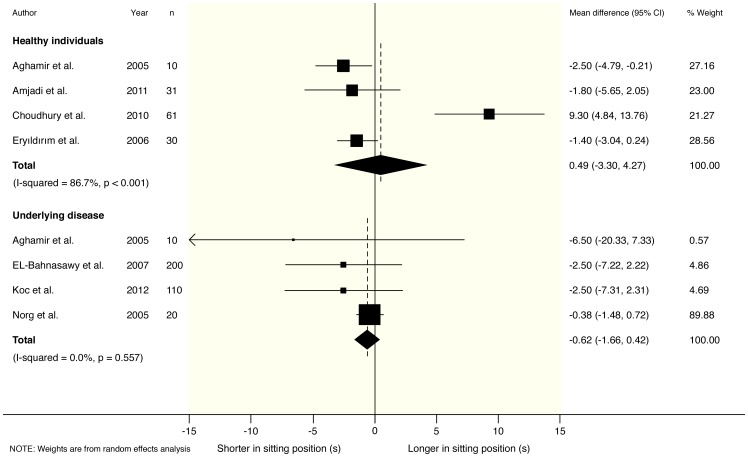
Forest plot from random effects meta-analysis on the difference in voiding time (TQ) in both healthy males and male patients with Lower Urinary Tract Symptoms (LUTS) in the sitting versus standing position.

**Figure 4:**
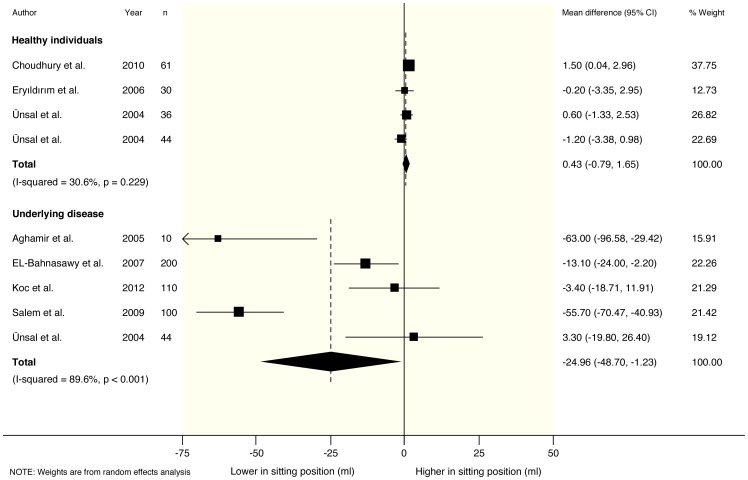
Forest plot from random effects meta-analysis on the difference in post-void residual volume (PVR) in both healthy males and male patients with Lower Urinary Tract Symptoms (LUTS) in the sitting versus standing position.

In healthy participants, no clear differences were found in any of the measured parameters for sitting versus standing position. Pooled mean differences from a random effects model were 0.18 ml/s (95% CI −1.67 to 2.02) for maximum urinary flow rate (Qmax), 0.49 s (95% CI −3.30 to 4.27) for voiding time (TQ) and 0.43 ml (95% CI −0.79 to 1.65) for post-void residual volume (PVR). Accompanying I^2^ statistics were 82% (p<0.001), 87% (p<0.001) and 31% (p = 0.229) respectively. These results are graphically depicted in figures 2–4.

### Meta-analysis of urodynamic parameters in patients with LUTS

For patients with LUTS, post-void residual volume (PVR) was found to be significantly decreased in the sitting position: −24.96 ml (95% CI −48.70 to −1.23) for PVR. In accordance Qmax was increased (1.23 ml/s; 95% CI −1.02 to 3.48), and TQ was reduced (−0.62 s; 95% CI −1.66 to 0.42) in sitting position, although these differences did not reach statistical significance. Accompanying I^2^ statistics were 90% (p<0.001), 98% (p<0.001), and 0% (p = 0.557) respectively. These results are graphically depicted in figures 2–4.

## Discussion

This study aims to determine the influence of body position during voiding on urodynamic parameters for both healthy males and men with LUTS by summarizing the scientific evidence for either the sitting or standing position. We found that in patients with LUTS the sitting position is associated with a trend towards a more favorable urodynamic profile: Qmax is increased, PVR is lowered and time spent urinating is shorter than in the standing position. In healthy males however, the results of our meta-analysis show no posture-related differences in any of the measured urodynamic parameters. We therefore conclude that for healthy men the debate on the standing versus sitting voiding posture cannot be settled by urodynamic arguments alone.

To our knowledge, this is the first systematic review and meta-analysis to assess urodynamics and posture-related changes. Three articles [3], [26], [34] provided a summarization in the form of a literature review, however a meta-analysis is of greater value in evidence-based clinical decision making. Apart from demonstrating a trend towards an improved urodynamic profile in LUTS patients, we unfortunately proved the accompanying heterogeneity as well. Consequently, some care should be taken in the interpretation of the results. Statistical analysis in order to identify the exact cause of the heterogeneity remains difficult in such a small number of studies. An arbitrary minimum number of ten studies is needed to perform funnel plot analysis and Egger tests to objectify publication bias. A lower number of studies results in diminished power and subsequently is prone to misinterpretation [35], [36]. It is not possible to pool our data in such a way that this requirement is met. For this reason, no additional tests to objectify bias of published studies were performed.

A possible explanation for heterogeneity in the data is the lack of standardization in the measurements of urodynamics. Influences of ejaculation [23], defecation [5], [18], intra-abdominal pressure [24], [25] and the setting in which measurements take place [21] affect urodynamics. Apart from the lack of standardization in measurements, the demography of the investigated population should be adequately described; for example, severity of LUTS was not properly objectified in all studies, allowing the possibility of inappropriate comparison. Another possible influencing factor is the accustomed voiding position or location: it is suggested that the mere change to a new position or urinating in a clinical research setting reflects negatively on urodynamic parameters, although no calculable data to perform a meta-analysis on this subject was presented [6], [8], [11], [14], [18], [26]–[28]. As seen in the risk of bias assessment, many studies did not take these influences into account. Methodological bias seems of lesser importance in this study. LUTS progresses relatively slowly, long-term follow-up is not required and a carry-over effect of the intervention (changing one's position) seems not likely. Therefore, the use of a cross-over design is an adequate study design, not prone to result in methodological bias [29], [37].

Several explanations for the described trend towards a better urodynamic profile in the sitting position are found in the included literature. The typical patients with LUTS/BPH are elderly males who are more prone to fall. It is suggested that the fear of falling while standing can result in involuntary contractions of the pelvic muscles to stabilize one's position [38]. Contraction of the pelvic muscles is related to a disturbed urinary flow [8]; relaxation of these muscles is better achieved by urinating in a sitting position and by supporting the feet in a comfortable position [39]–[41]. Also, muscle tension in the medial and anterior compartments of the hip is decreased in the sitting position [7]. These muscles, if actively contracted, increase the contractility of the pelvic floor muscles. Furthermore, contraction of the pelvic floor musculature inhibits the activity of the detrusor urinae muscle [42]. Contraction of the detrusor is needed for urination, consequently increased activity of the pelvic floor musculature negatively influences urodynamics [5], [8], [25], [28].

Besides muscle contraction, urination in the sitting position is also associated with defecation. During sitting and especially during defecation, the intra-abdominal pressure rises, influencing urodynamics [5], [28]. Furthermore, innervation of the anal sphincter and the pelvic floor musculature both arise from the sacral plexus (S2–4). It is suggested that the contraction of the anal sphincter is associated with an increased activity of the pelvic floor muscles due to this common innervation [5], [7]. The desire not to lose defecation or flatus in standing position, especially in public conveniences, can thus lead to increased activity of the pelvic floor musculature and consequently impaired micturition.

The urodynamic profile of LUTS patients is one of decreased Qmax and increased TQ and PVR, a pattern that is known to increase the risk of certain urological complications, e.g. cystitis and bladder stones. We found a decrease in PVR and TQ, while Qmax was increased in the sitting position. Extrapolating this trend, the alleviation of this impaired urodynamic profile in this group may possibly reduce complaints as well as the incidence of cystitis and bladder stones [5], [8], [11]–[14]. The increase in maximum urinary flow rate (Qmax) of 1.23 ml/s may seem low. However, compared to existing medical treatments of LUTS, the increase is relevant despite not reaching statistical significance. A meta-analysis of the efficacy of four alpha-1 blockers (Alfuzosin, Tamsulosin, Terazosin, and Doxazosin) found an increase in Qmax by 1.32 ml/sec (95% CI: 1.07 to 1.57) [16]. We did not find meta-analyses on the influence of these pharmaceutics on TQ or PVR. It is intriguing that the effect of changing to a sitting voiding posture – a simple intervention without any serious side effects – approaches the effect of conventional pharmacological treatment of LUTS. However, it should not be regarded as the sole therapy instead of pharmacological treatment, perhaps both interventions combined could have a synergistic effect on urodynamics in the management of LUTS.

## Conclusion

In patients with LUTS, an improved urodynamic profile approaching the effect of alpha-blockers is found in the sitting position. Incorporating the positive effect of this voiding position in the management of LUTS could have a synergistic effect on improvement of urodynamics in this group of patients. As no effect of changing voiding position in healthy males was found, our study does not translate into a medically preferable position for healthy males to urinate in.

### Patient summary

In this report we've looked at the influences of changing urination posture on the maximum urine flow, the time spent voiding and the amount of urine that is left in the bladder. We conclude that the sitting posture is the best position for men with urination problems, e.g. due to an enlarged prostate to urinate in, whereas no difference was found in healthy men. This is clinically important, because residual urine may result in complications such as cystitis and bladder stones.

### Take home messages

1. Comparing the standing with the sitting position, for patients with Lower Urinary Tract Symptoms (LUTS) the sitting voiding position is preferable to the standing. However, there is medically no superior posture for healthy men to urinate in.
2. The positive influence of urinating in the sitting position approaches the effects of standard pharmacological therapy in LUTS patients.

## Supporting Information

Table S1 **PRISMA checklist.**
(DOCX)

Appendix S1 **Search strategies.**
(DOCX)
