# Supplementary material for: Urinating Standing versus Sitting: Position Is of Influence in Men with Prostate Enlargement. A Systematic Review and Meta-Analysis
Source: PLoS One. 2014 Jul 22;9(7):e101320. doi: 10.1371/journal.pone.0101320 (PMC4106761; doi:10.1371/journal.pone.0101320)
Supplement: Appendix S1 — Search strategies. (DOCX) [file pone.0101320.s002.docx]

**PubMed**

409 results

Date: 25-04-2013

(("Posture"[majr] OR posture[ti] OR position[ti] OR standing[ti] OR stand[ti] OR sitting[ti] OR sit[ti] OR crouching[ti] OR crouched[ti] OR crouch[ti] OR squatting[ti] OR "body position"[ti] OR "Biomechanics"[majr]) AND (urinating[ti] OR urinate[ti] OR urination[ti] OR "Urination"[majr] OR "Urination Disorders"[majr] OR "Urinary Bladder/physiology"[majr] OR "Urodynamics"[majr] OR urodynamic[ti] OR urodynamics[ti] OR voiding[ti] OR Micturition[ti] OR Mictur*[ti] OR uroflowmetry[ti] OR uroflowmetric[ti] OR uroflow[ti] OR uroflow*[ti] OR ((urine[ti] OR "Urine"[majr]) AND (discharge[ti] OR discharg*[ti] OR volume[ti] OR flow rate[ti] OR flow[ti])) OR "Diuresis"[majr] OR Diuresis[ti] OR (bladder[ti] AND emptying[ti]) OR "Urinary bladder"[majr] OR "urinary bladder"[ti] OR "Urine"[majr] OR urine[ti] OR "Urography"[majr] OR urography[ti] OR "urinary retention"[majr] OR "urinary retention"[ti] OR "urination disorders"[majr] OR "urethra"[majr] OR urethra[ti] OR ("Urologic Diseases"[majr] NOT "Kidney Diseases"[majr])) NOT ("female"[mesh] NOT "male"[mesh])) OR ("voiding position" NOT ("female"[mesh] NOT "male"[mesh])) NOT ("Animals"[mesh] NOT "Humans"[mesh])

<http://www.ncbi.nlm.nih.gov/entrez/query.fcgi?otool=leiden>

**Embase (OVID-version)**

168 results

Date: 25-04-2013

((exp *body position/ OR posture.ti OR position.ti OR standing.ti OR stand.ti OR sitting.ti OR sit.ti OR crouching.ti OR crouched.ti OR crouch.ti OR squatting.ti OR "body position".ti OR *Biomechanics/) AND (exp *bladder function/ OR urinating.ti OR urinate.ti OR urination.ti OR exp *micturition disorder/ OR urodynamic.ti OR urodynamics.ti OR voiding.ti OR Micturition.ti OR Mictur*.ti OR uroflowmetry.ti OR uroflowmetric.ti OR uroflow.ti OR uroflow*.ti OR ((urine.ti OR *Urine/) AND (discharge.ti OR discharg*.ti OR volume.ti OR flow rate.ti OR flow.ti)) OR exp *Diuresis/ OR Diuresis.ti OR (bladder.ti AND emptying.ti) OR exp *bladder/ OR "urinary bladder".ti OR *Urine/ OR urine.ti OR exp *Urography/ OR urography.ti OR *urine retention/ OR "urinary retention".ti OR exp *Urethra/ OR urethra.ti OR (exp *urinary tract disease/ NOT exp *Kidney Diseases/)) AND (exp male/ OR (male OR men OR man OR boy OR boys).mp)) OR ("voiding position".mp NOT (exp male/ OR (male OR men OR man OR boy OR boys).mp)) AND exp Human/

<http://ovidsp.ovid.com/ovidweb.cgi?T=JS&PAGE=main&MODE=ovid&D=oemezd>

**PubMed Central**

33 results

Date: 25-04-2013

((("voiding position"[all fields] OR "micturition posture"[all fields] OR "toileting behavior"[all fields] OR "toileting behaviour"[all fields] OR "toileting behaviors"[all fields] OR"Posture"[majr] OR posture[Ti] OR position[Ti] OR standing[Ti] OR stand[Ti] OR sitting[Ti] OR sit[Ti] OR crouching[Ti] OR crouched[Ti] OR crouch[Ti] OR squatting[Ti] OR "body position"[Ti] OR "Biomechanics"[majr]) AND (urinating[Ti] OR urinate[all fields] OR urination[Ti] OR "Urination"[majr] OR "Urination Disorders"[majr] OR "Urinary Bladder/physiology"[majr] OR "Urodynamics"[majr] OR urodynamic[Ti] OR urodynamics[Ti] OR voiding[Ti] OR Micturition[Ti] OR uroflowmetry[Ti] OR uroflowmetric[Ti] OR uroflow[Ti] OR uroflow*[Ti] OR ((urine[Ti] OR "Urine"[majr]) AND (discharge[Ti] OR volume[Ti] OR "flow rate" [Ti] OR flow[Ti])) OR "Diuresis"[majr] OR Diuresis[Ti] OR (bladder[Ti] AND emptying[Ti]) OR "Urinary bladder"[majr] OR "urinary bladder"[Ti] OR "Urine"[majr] OR urine[Ti] OR "Urography"[majr] OR urography[Ti] OR "urinary retention"[majr] OR "urinary retention"[Ti] OR "urination disorders"[majr] OR "urethra"[majr] OR urethra[Ti] OR ("Urologic Diseases"[majr] NOT "Kidney Diseases"[majr])) NOT ("female"[mesh] NOT "male"[mesh])) OR ("voiding position" NOT ("female"[mesh] NOT "male"[mesh])) NOT ("Animals"[mesh] NOT "Humans"[mesh]))

<http://www.ncbi.nlm.nih.gov/pmc/>

**Web of Science**

186 results

Date: 25-04-2013

TI=("voiding position" OR "micturition position" OR "urinating position" OR "bladder emptying position" OR "micturition position" OR "voiding posture" OR "micturition posture" OR "urinating posture" OR "bladder emptying posture" OR "toileting behavior" OR "toileting behaviour" OR "toileting behaviors" OR "toileting behaviours" OR (("Standing" OR "Sitting" OR "seated" OR "Squatting" OR "Crouching" OR "Recumbent" OR "Prone" OR "Supine" OR "Lateral") AND ( "Uroflow" OR "Uroflowmetry" OR "Uroflowmetric" OR "Voided" OR "Voiding" OR "bladder" OR "Micturition")) OR (("position" OR "positions") AND ("Urine" OR "Urinary" OR "Uroflow" OR "Uroflowmetry" OR "Uroflowmetric" OR "Voided" OR "Voiding" OR "bladder" OR "Micturition"))) NOT TS= (animal OR position paper OR meeting abstract OR conference abstract)

<http://isiknowledge.com/wos>

**Cochrane Library**

216 results

Date: 25-04-2013

Title, abstract, keywords

("voiding position" OR "micturition position" OR "urinating position" OR "bladder emptying position" OR "micturition position" OR "voiding posture" OR "micturition posture" OR "urinating posture" OR "bladder emptying posture" OR "toileting behavior" OR "toileting behaviour" OR "toileting behaviors" OR "toileting behaviours" OR (("Standing" OR "Sitting" OR "seated" OR "Squatting" OR "Crouching" OR "Recumbent" OR "Prone" OR "Supine" OR "Lateral") AND ( "Uroflow" OR "Uroflowmetry" OR "Uroflowmetric" OR "Voided" OR "Voiding" OR "bladder" OR "Micturition")) OR (("position" OR "positions") AND ("Uroflow" OR "Uroflowmetry" OR "Uroflowmetric" OR "Voided" OR "Voiding" OR "bladder" OR "Micturition")) NOT("animal" OR "meeting abstract" OR "conference abstract" OR "position paper"))

<http://www.thecochranelibrary.com/view/0/index.html>

**CINAHL**

304 results

Date: 25-04-2013

All text

TX ( (voiding position OR micturition position OR urinating position OR bladder emptying position OR micturition position OR voiding posture OR micturition posture OR urinating posture OR bladder emptying posture OR toileting behavior OR toileting behaviour OR toileting behaviors OR toileting behaviours OR ((Standing OR Sitting OR seated OR Squatting OR Crouching OR Recumbent OR Prone OR Supine OR Lateral) AND (Uroflow OR Uroflowmetry OR Uroflowmetric OR Voided OR Voiding OR bladder OR Micturition)) OR ((position OR positions) AND (Uroflow OR Uroflowmetry OR Uroflowmetric OR Voided OR Voiding OR bladder OR Micturition))) ) NOT (TX (animal OR position paper OR meeting abstract OR conference abstract))

<http://search.ebscohost.com/login.aspx?authtype=ip,uid&profile=lumc&defaultdb=cin20>

**PsycINFO**

97 results

Date: 25-04-2013

All text

TX ( (voiding position OR micturition position OR urinating position OR bladder emptying position OR micturition position OR voiding posture OR micturition posture OR urinating posture OR bladder emptying posture OR toileting behavior OR toileting behaviour OR toileting behaviors OR toileting behaviours OR ((Standing OR Sitting OR seated OR Squatting OR Crouching OR Recumbent OR Prone OR Supine OR Lateral) AND (Uroflow OR Uroflowmetry OR Uroflowmetric OR Voided OR Voiding OR bladder OR Micturition)) OR ((position OR positions) AND (Uroflow OR Uroflowmetry OR Uroflowmetric OR Voided OR Voiding OR bladder OR Micturition))) ) NOT (TX (animal OR position paper OR meeting abstract OR conference abstract))

<http://search.ebscohost.com/login.aspx?authtype=ip,uid&profile=lumc&defaultdb=psyh>

**Academic Search Premier**

via CINAHL or PsycINFO by altering the option “Choose Database”

277 results

Date: 25-04-2013

"voiding position" OR "micturition position" OR "urinating position" OR "bladder emptying position" OR "micturition position" OR "voiding posture" OR "micturition posture" OR "urinating posture" OR "bladder emptying posture" OR "toileting behavior" OR "toileting behaviour" OR "toileting behaviors" OR "toileting behaviours" OR (("Standing" OR "Sitting" OR "seated" OR "Squatting" OR "Crouching" OR "Recumbent" OR "Prone" OR "Supine" OR "Lateral") AND ("Uroflow" OR "Uroflowmetry" OR "Uroflowmetric" OR "Voided" OR "Voiding" OR "Micturition")) OR (("position" OR "positions") AND ("Uroflow" OR "Uroflowmetry" OR "Uroflowmetric" OR "Voided" OR "Voiding" OR "Micturition")) NOT ("animal" OR "meeting abstract" OR "conference abstract" OR "position paper")

**ScienceDirect**

124 results

Date: 25-04-2013

(voiding position OR micturition position OR urinating position OR bladder emptying position OR micturition position OR voiding posture OR micturition posture OR urinating posture OR bladder emptying posture OR toileting behavior OR toileting behaviour OR toileting behaviors OR toileting behaviours OR ((Standing OR Sitting OR seated OR Squatting OR Crouching OR Recumbent OR Prone OR Supine OR Lateral) AND (Uroflow OR Uroflowmetry OR Uroflowmetric OR Voided OR Voiding OR bladder OR Micturition)) OR ((position OR positions) AND (Urine OR Urinary OR Uroflow OR Uroflowmetry OR Uroflowmetric OR Voided OR Voiding OR bladder OR Micturition))AND NOT (animal OR position paper OR meeting abstract OR conference abstract))

<http://www.sciencedirect.com/science?_ob=MiamiSearchURL&_method=requestForm&_temp=all_boolSearch.tmpl&_acct=C000026638&_version=1&_urlVersion=1&_userid=530453&md5=d44bd9fa9076bb9b258a588b309be1e3>

**SpringerLink**

13 results

Date: 25-04-2013

"where the title contains"

((("voiding position" OR "micturition position" OR "urinating position" OR "bladder emptying position" OR "micturition position" OR "voiding posture" OR "micturition posture" OR "urinating posture" OR "bladder emptying posture" OR "toileting behavior" OR "toileting behaviour" OR "toileting behaviors" OR "toileting behaviours" OR "Standing" OR "Sitting" OR "seated" OR "Squatting" OR "Crouching" OR "Recumbent" OR "Prone" OR "Supine" OR "Lateral") AND ("Uroflow" OR "Uroflowmetry" OR "Uroflowmetric" OR "Voided" OR "Voiding" OR "bladder" OR "Micturition")) AND NOT (animal OR position paper OR meeting abstract OR conference abstract))

<http://springerlink.metapress.com/app/home/search-itations.asp?wasp=3pvxje0mwm6qxm8d2hw3>

**Wiley Online Library**

41 results

Date: 25-04-2013

(("voiding position" OR "micturition position" OR "urinating position" OR "bladder emptying position" OR "micturition position" OR "voiding posture" OR "micturition posture" OR "urinating posture" OR "bladder emptying posture" OR "toileting behavior" OR "toileting behaviour" OR "toileting behaviors" OR "toileting behaviours" OR "Standing" OR "Sitting" OR "seated" OR "Squatting" OR "Crouching" OR "Recumbent" OR "Prone" OR "Supine" OR "Lateral") AND ("Urine" OR "Urinary" OR "Uroflow" OR "Uroflowmetry" OR "Uroflowmetric" OR "Voided" OR "Voiding" OR "bladder" OR "Micturition")) in Article Titles

<http://www3.interscience.wiley.com/cgi-bin/simplesearch>

**Lippincott-Williams&Wilkins (Journals@Ovid Full Text)**

184 results

Date: 25-04-2013

("voiding position" OR "micturition position" OR "urinating position" OR "bladder emptying position" OR "micturition position" OR "voiding posture" OR "micturition posture" OR "urinating posture" OR "bladder emptying posture" OR "toileting behavior" OR "toileting behaviour" OR "toileting behaviors" OR "toileting behaviours").af OR ((("Standing" OR "Sitting" OR "seated" OR "Squatting" OR "Crouching" OR "Recumbent" OR "Prone" OR "Supine" OR "Lateral") AND ("Urine" OR "Urinary" OR "Uroflow" OR "Uroflowmetry" OR "Uroflowmetric" OR "Voided" OR "Voiding" OR "bladder" OR "Micturition")) OR (("position" OR "positions") AND ("Uroflow" OR "Uroflowmetry" OR "Uroflowmetric" OR "Voided" OR "Voiding" OR "bladder" OR "Micturition"))NOT(animal OR position paper OR meeting abstract OR conference abstract)).ti

<http://ovidsp.ovid.com/ovidweb.cgi?T=JS&PAGE=main&MODE=ovidclassic&D=ovft>

**Highwire**

58 results

Date: 25-04-2013

("voiding position" OR "micturition position" OR "urinating position" OR "bladder emptying position" OR "micturition position" OR "voiding posture" OR "micturition posture" OR "urinating posture" OR "bladder emptying posture" OR "toileting behavior" OR "toileting behaviour" OR "toileting behaviors" OR "toileting behaviours")

<http://highwire.stanford.edu/>

**Google Scholar**

248-241 results, 244 exported.

Date: 25-04-2013

“voiding position" OR "micturition position" OR "urinating position" OR "bladder emptying position" OR "micturition position" OR "voiding posture" OR "micturition posture" OR "urinating posture" OR "bladder emptying posture"

<http://scholar.google.com/>
